# Photoprotection and skin cancer awareness in kidney transplant recipients living with HIV: a single-centre cross-sectional study

**DOI:** 10.1093/skinhd/vzaf016

**Published:** 2025-05-12

**Authors:** Fatima Ali, Antonia Cronin

**Affiliations:** Department of Nephrology and Transplantation, Guy’s and St Thomas’ Hospitals NHS Trust, London, UK; King’s College London, London, UK; Department of Nephrology and Transplantation, Guy’s and St Thomas’ Hospitals NHS Trust, London, UK; King’s College London, London, UK

## Abstract

**Background:**

Immunosuppression medication is an important risk factor for skin cancer in kidney transplant recipients (KTR). Both HIV-associated immunodeficiency and immunosuppression required for kidney transplantation increase skin cancer rates. Kidney transplant recipients living with HIV (KTRLHIV) must take steps to minimize their risk of skin cancer through prevention.

**Objectives:**

To conduct a cross-sectional study to investigate photoprotection knowledge and practices, and skin cancer awareness in KTRLHIV compared with a matched cohort of HIV-negative KTR.

**Methods:**

Our study, conducted at a tertiary UK HIV kidney transplantation centre, utilized a validated sun photoprotection and skin cancer awareness questionnaire either online or in person. The KTRLHIV cohort (*n* = 27) and HIV-negative KTR cohort (*n* = 25) were matched for age, sex, ethnicity and years since transplant.

**Results:**

Only 60% of KTRLHIV had been seen by a dermatologist, compared with 81% in the matched KTR cohort. Sun protection advice was received by 52% of KTRLHIV, significantly lower than the 80% in the matched cohort (*P* = 0.03), primarily sourced from nephrologists or dermatologists. KTRLHIV exhibited lower overall sunscreen use (33% vs. 60%, *P* = 0.05), fewer daily users (22% vs. 27%), lower utilization of sun protection factor > 25 (78% vs. 100%) and lower use on all exposed areas (67% vs. 87%). Sun protection behaviours were also suboptimal in KTRLHIV compared with the matched cohort, with regard to avoiding direct sun exposure (*P* = 0.003), and dressing to protect from the sun (*P* < 0.001).

**Conclusion:**

Our findings reveal lower rates of skin cancer protection advice for KTRLHIV compared with matched HIV-negative KTR, likely translating into decreased sunscreen use and suboptimal sun protection behaviours. Addressing this disparity through skin cancer prevention, self-skin examination education and improved dermatology referrals is necessary.

## Teaser

This study investigates photoprotection practices and skin cancer awareness among kidney transplant recipients living with HIV (KTRLHIV) compared with HIV-negative kidney transplant recipients. Findings reveal lower rates of dermatology referrals and skin cancer protection advice, and suboptimal sunscreen use in KTRLHIV, highlighting the need for targeted educational interventions and improved dermatological care for this high-risk group.

**What is already known about this topic?**

- Kidney transplant recipients living with HIV (KTRLHIV) face an increased risk of skin cancer.
- There is a paucity of research on photoprotection and skin cancer awareness in KTRLHIV, creating a significant knowledge gap.

**What does this study add?**

- This study provides comprehensive insights into KTRLHIV photoprotection practices and skin cancer awareness, comparing them with matched HIV-negative kidney transplant recipients.
- This research identifies a notable disparity in skin cancer protection advice, sunscreen use and photoprotection behaviours between KTRLHIV and a matched HIV-negative cohort.

Compared with the general population, kidney transplant recipients (KTR) are at considerably higher risk of developing skin cancer. Incidence rates are estimated to be between 10% and 45% at 10 years post-transplant for nonmelanoma skin cancers. In particular, there is a 65- to 250-fold increase in the incidence and prevalence of squamous cell carcinoma (SCC) in this group when compared with an aged-matched general population.^1^ This increased risk stems principally from immunosuppressive medication type and duration, in addition to known pretransplant risk factors including skin phototype, previous skin cancers and ultraviolet (UV) ­exposure.

HIV is also associated with an increased risk of developing skin cancer. Individuals living with HIV have a twofold increased risk of basal cell carcinoma (BCC) and a fivefold increased risk of SCC compared with the general population, with the risk commensurate to the level of disease control.^2,3^ In addition, the incidence of melanoma is reported to be 2.6 times higher.^2,3^ This is postulated to be due to a combination of immunosuppression and altered DNA repair mechanisms.^4^ Due to the effectiveness of highly active antiretroviral therapy (HAART), the incidences of cutaneous non-AIDS-defining cancers (e.g. BCC, SCC and melanoma) have surpassed rates of AIDS-defining cancers such as Kaposi sarcoma.^5^ However, solid organ transplant recipients face a 125-fold higher risk of developing Kaposi sarcoma, while patients with HIV face a 451-fold higher risk compared with the general population.^6^

The number of KTR living with HIV (KTRLHIV) in the UK is currently small. Despite a relatively small cohort size in our study, it constitutes a meaningful proportion of this unique population, representing one of the largest groups of KTRLHIV in the UK.^7^ To contextualize this, a 2024 population-based study conducted in Ontario identified a cohort of 21 KTRLHIV among a total of 5334 transplant recipients throughout the province.^8^

Advances in HAART have allowed for better viral control, contributing to HIV becoming a manageable chronic disease with improved life expectancy. Hence, more patients with HIV are developing long-term conditions including chronic kidney disease.^9^ We can anticipate increasing demand for kidney transplantation in those living with HIV in the coming years. Therefore, further research and tailored skin cancer education for this group are necessary. Currently, no studies have explored educational photoprotection or self-skin examination advice in patients living with HIV.

The potential dual immunosuppressive effect from transplant antirejection immunosuppression and HIV means that KTRLHIV are likely to be at even greater risk of developing skin cancer post-transplant.^10^ However, there is a paucity of research surrounding photoprotection and skin cancer awareness in KTRLHIV. Given the increased risk, tailored preventive and photoprotective strategies for this high-risk population are likely to be necessary.^11^

We conducted a cross-sectional study to investigate photoprotection knowledge, practices and skin cancer awareness in KTRLHIV compared with a matched cohort of HIV-negative KTR. Our study employed validated questionnaires adapted from Ismail *et al.*^12^ to assess­ photo­protection advice, employed behaviours, sunscreen use and knowledge regarding skin cancer and photoprotective measures. By comparing these factors between KTRLHIV and HIV-negative KTR, we aimed to identify any potential disparities in awareness and practice, informing targeted interventions to optimize skin cancer prevention.

## Patients and methods

### Study design

The study employed a single-centre cross-sectional design to investigate photoprotection knowledge and practices, and skin cancer awareness in KTRLHIV compared with a matched cohort of HIV-negative KTR. This design allowed for a snapshot comparison of both populations at a single point in time.

### Participants

All KTRLHIV (*n* = 29) were identified through our tertiary kidney transplant centre database. Two patients chose not to participate in the study, allowing for a total of 27 participants.

Initially, 27 HIV-negative KTR at our tertiary centre were matched using a propensity score-matching algorithm based on age, sex, ethnicity and years since transplant. However, one matched participant withdrew, and one did not complete the questionnaire, allowing for 25 HIV-negative KTR. Despite this, the two cohorts remained closely matched. This procedure ensured comparable demographic and transplant-related characteristics between the two groups.

### Data collection

Participants completed validated questionnaires either online or in person during a 4-week period in April 2023. These questionnaires assessed photoprotection and skin cancer awareness, and were adapted from Ismail *et al*.^12^ The full questionnaire is provided in Appendix S1 (see Supporting Information). The questionnaire included questions relating to awareness of increased skin cancer risk, skin cancer awareness advice, photoprotection advice and behaviours. The results of the questionnaire were uploaded to the patient’s electronic health record.

### Statistical analysis

Descriptive statistics were used to summarize participant characteristics and questionnaire responses. χ^2^ or Fisher’s exact tests were employed to compare categorical variables between the KTRLHIV and HIV-negative KTR groups. Continuous variables were assessed using independent *t*-tests or Mann–Whitney *U* tests, depending on data normality. Thematic analysis was completed for qualitative results using NVivo software (https://lumivero.com/products/nvivo/) and visualizations were created using RStudio version 2022.07.1+554 (https://dailies.rstudio.com/version/2022.07.1+554/). Statistical significance was set at *P* < 0.05.

## Results

### Baseline characteristics

On comparison of baseline characteristics of the two cohorts, there was no significant difference in mean age, sex, ethnicity or years since transplant. The median age of KTRLHIV was 55 years (range 43–74) and for HIV-negative KTR it was 54 years (range 44–72). There was a total of 33 (64%) men, including 17 (63%) KTRLHIV and 16 (64%) HIV-negative KTR (*P* > 0.99). The majority of participants were Black (*n* = 36/52; 69%), including 19 (70%) KTRLHIV and 17 (68%) HIV-negative KTR. There were by 14 (27) White participants, equally divided between KTRLHIV (*n* = 7; 26%) and HIV-negative KTR (*n* = 7; 28%). There was one Asian participant in each cohort, representing 4% of KTRLHIV and 4% of HIV-negative KTR. There was no significant difference in ethnicity between the cohorts (*P* = 0.98).

The mean (SD) time since transplant for KTRLHIV was 7.4 (SD 6.0) years and for HIV-negative KTR it was 7.7 (6.3) years (*P* = 0.86). All KTRLHIV had an undetectable viral load at the point of data collection. Most patients were receiving standard triple immunosuppressive therapy with prednisolone, tacrolimus and mycophenolate mofetil, with no significant difference between KTRLHIV and HIV-negative KTR. Mean estimated glomerular filtration rate in KTRLHIV vs. matched HIV-negative KTR was 34 and 43 mL min^−1^ (*P* = 0.05). Participant demographics are shown in Table 1.

**Table 1:** Baseline characteristics of kidney transplant recipients living with HIV (KTRLHIV) and HIV-negative kidney transplant recipients (KTR)

	KTRLHIV (*n* = 27)	HIV-negative KTR (*n* = 25)	*P*-value	Overall (*n* = 52)
Sex				
Female	10 (37)	9 (36)	> 0.99	19 (37)
Male	17 (63)	16 (64)		33 (64)
Age (years)				
Mean (SD)	55.6 (8.8)	55.5 (7.6)	0.96	55.6 (8.2)
Median (range)	54.0 (43.0–74.0)	55.0 (45.0–68.0)		55.0 (43.0–74.0)
Ethnicity				
Asian	1 (4)	1 (4)	0.98	2 (4)
Black	19 (70)	17 (68)		36 (69)
White	7 (26)	7 (28)		14 (27)
Years since transplant				
Mean (SD)	7.4 (6.0)	7.7 (6.3)	0.86	7.5 (6.1)
Median (range)	4.6 (0.7–23.6)	5.5 (0.7–23.7)		5.3 (0.7–23.7)

Data are presented as *n* (%) unless otherwise stated. No significant differences were found between the groups with regard to sex, age, ethnicity and years since transplant.

### Specialist review

**Figure 1:**
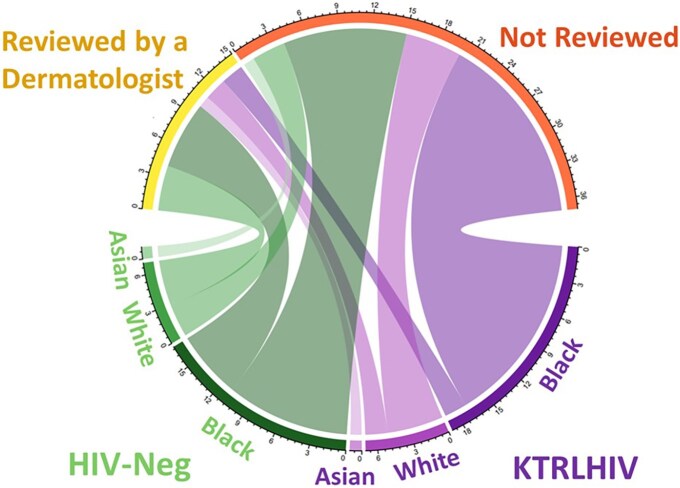
Chord diagram illustrating dermatologist review among kidney transplant recipients living with HIV (KTRLHIV) and HIV-negative kidney transplant recipients (KTR) by ethnicity (Black, White, Asian). Each band along the lower half represents an ethnicity and its width represents the number of participants. Ribbons connect these bands to the upper bands, which represent dermatologist review outcomes: reviewed or not reviewed. The diagram highlights the distribution of dermatologist reviews across HIV status and ethnicity.

Patients were asked whether they had been reviewed by a skin doctor. The results are shown in Figure 1. Eighty-one percent (*n* = 22) of KTRLHIV reported not being reviewed by a skin doctor in the last year vs. 60% (*n* = 15) of the matched KTR cohort (*P* = 0.09).

Of the 22 KTRLHIV who reported they had not had a dermatology review, 59% (*n* = 13) were men with a mean age of 53.8 years and were, on average, 5.9 years post-­transplant; 77% (*n* = 17) were Black and 23% (*n* = 5) were White. Of the 15 matched HIV-negative KTR who reported they had not had a dermatology review, 53% (*n* = 8) were men, with a mean age of 56.5 years and were, on average, 7.0 years post-transplant 73% (*n* = 11) were Black, 20% (*n* = 3) White and 7% were Asian (*n* = 1). A relatively greater proportion of Black patients in both groups had lower rates of reported dermatology review.

In comparison, 85% (*n* = 23) of KTRLHIV had visited a nephrologist at least three times in the last year, and the remaining 15% (*n* = 4) had visited a nephrologist twice in the last year. All of the HIV-negative KTR participants (*n* = 25; 100%) had visited a nephrologist at least three times in the last year.

### Skin cancer awareness and photoprotection advice

Questions included whether participants had received information relating to skin cancer awareness and photoprotection advice, and, if so, who had provided this information and whether it was provided before/after their transplant. Additionally, participants were asked how many times this information was provided and whether any written advice was provided.

Significantly lower rates of receiving skin cancer awareness and photoprotection advice was reported in KTRLHIV. Advice was reported to have been given in 52% (*n* = 14/27) of KTRLHIV vs. 80% (*n* = 20/25) of the matched HIV-negative KTR cohort (*P* = 0.03). In both groups, all participants who were not provided with advice were either Black or Asian (*P* = 0.004). Of the participants who did receive skin cancer awareness and photoprotection advice, only 50% (*n* = 7/14) of KTRLHIV and 55% (*n* = 11/20) of matched KTR recalled receiving this before their transplant.

In relation to the number of times skin cancer and ­photoprotection advice was received, of the KTRLHIV who received advice (*n* = 14), 43% (*n* = 6/14) received this once, 50% (*n* = 7/14) twice and 7% (*n* = 1/14) more than twice. In comparison, in the matched HIV-negative KTR cohort (*n* = 20), 20% (*n* = 4/20) received advice once, 55% (*n* = 11/20) twice and 25% (*n* = 5/20) more than twice (*P* = 0.22). Also, a significant difference was noted when comparing whether written advice was received. Only 29% (*n* = 4/14) of KTRLHIV compared with 65% (*n* = 13/20) of matched HIV-negative KTR received written information (*P* = 0.04). The source of advice in the majority of both KTRLHIV and matched HIV-negative KTR cohorts was the participants’ nephrologist [46% (*n* = 11/24) and 52% (*n* = 16/31), respectively] or dermatologist [21% (*n* = 5/24) and 35% (*n* = 11/31), respectively]. KTRLHIV and HIV-negative KTR also reported receiving information from their kidney nurse [29% (*n* = 7/24) and 13% (*n* = 7/24), respectively] and in 4% (*n* = 1/24) of KTRLHIV the source of information was the media. No participants reported receiving skin cancer or photoprotection advice from their general practitioner.

### Sunscreen use and photoprotection behaviours

Thirty-three per cent (*n* = 9/27) of KTRLHIV and 60% (*n* = 15/25) of matched KTR reported using sunscreen (*P* = 0.05). The majority of participants of both cohorts [78% (n = 7/9) and 73% (n = 11/15), respectively] reported only using sunscreen when it is sunny, and only a small proportion reported daily use [22% (*n* = 2/9) and 27% (*n* = 4/15), respectively]. However, if sunscreen was used, the majority [78% (*n* = 7/9) and 100% (*n* = 15/15), respectively] reported using sun protection factor 25 or greater and using sunscreen on all exposed areas [67% (*n* = 6/9) and 87% (*n* = 13/15), respectively]. Of those who reported not ever wearing sunscreen, all but three participants (two White participant and one Asian participant) in the KTRLHIV cohort were Black, whereas all in the matched HIV-negative group were Black.

Photoprotection behaviours were assessed through questions on avoiding direct sun exposure and dressing to protect against the sun.

**Figure 2:**
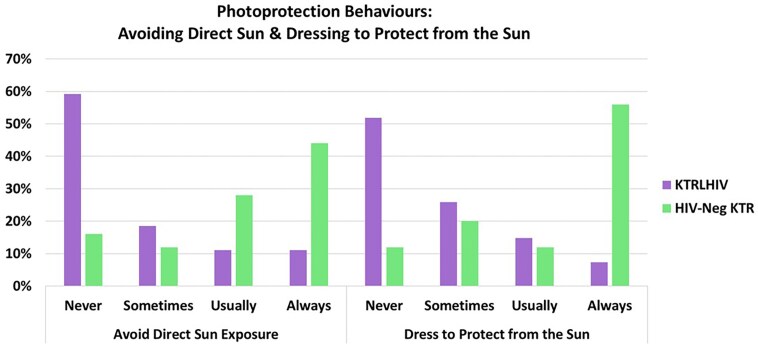
Photoprotection behaviours in kidney transplant recipients living with HIV (KTRLHIV) and HIV-negative kidney transplant recipients (KTR).

KTRLHIV, compared with matched KTR, displayed suboptimal photoprotection behaviours with regard to avoiding direct sun exposure, as indicated by an overall significant χ^2^ value χ^2^ (degrees of freedom = 3, *n* = 52) = 13.81, yielding a *P-*value of 0.003 (Figure 2). Particularly during the summer, 59% (*n* = 16/27) of KTRLHIV reported never avoiding direct sun exposure vs. 16% (*n* = 4/25) of KTR (*P* = 0.001). Only 11% (*n* = 3/27) of KTRLHIV vs. 44% (*n* = 11/25) KTR reported always avoiding direct sun exposure in summer (*P* = 0.008).

Furthermore, when compared with the matched KTR group, KTRLHIV were found to have significantly reduced photoprotection practices with regard to dressing to protect from the sun (*P* < 0.001) (Figure 2). These findings include a significant difference in KTRLHIV vs. KTR never dressing to protect themselves from the sun [52% (*n* = 14/27) vs. 12% (*n* = 3/25)] and those who always dress to protect from the sun [7% (*n* = 2/27) vs. 56% (*n* = 14/25)]. Notably, of those who reported never avoiding direct sun or dressing to protect from the sun, all but three participants (two White participants and one Asian participant) in the KTRLHIV cohort were Black, whereas all in the matched KTR group were Black.

### Skin cancer awareness

Regarding awareness of increased skin cancer risk compared with the general population, only 48% (*n* = 13/27) of KTRLHIV were aware of their increased risk of skin cancer vs. 64% (*n* = 16/25) of KTR (*P* = 0.54). Of the participants who were unaware of their increased risk of skin cancer, 43% (*n* = 6/14) in the KTRLHIV cohort were men vs. 56% (*n* = 5/9) in the matched KTR cohort. The mean age was 58 years in both groups, and they were 5.8 years and 5.3 years post-transplant, respectively. KTRLHIV who were unaware of their increased skin cancer risk were all Black (*n* = 13) or Asian (*n* = 1), compared with KTR who were Black (*n* = 8) and White (*n* = 1).

Qualitative thematic analysis was performed on the output of the open questions on understanding why photoprotection, skin cancer and self-skin checking are important. Themes included correct associations of medication-related immunosuppression, age, UV exposure and lighter skin with skin cancer. However, incorrect associations were also identified, including believing skin cancer is caused by heat, poor kidney function or a kidney transplant reaction with skin. Few KTRLHIV (*n* = 10) and KTR (*n* = 6) knew that photoprotection, skin cancer and self-skin checking was important but were unsure of the reasons why. Also, Black patients in both groups reported that they considered they were protected from any skin cancers and had no need to perform skin checks.

## Discussion

We have found statistically significant lower knowledge of skin cancer awareness and photoprotection in KTRLHIV compared with matched HIV-negative KTR. Lower rates of skin cancer protection advice may have resulted in lower rates of sunscreen use and poorer photoprotection behaviours in KTRLHIV. Despite statistically significant lower rates of knowledge and understanding in KTRLHIV, both groups displayed unsatisfactory skin cancer awareness/photoprotection behaviours.

In our study, KTRLHIV were less likely to have received dermatologist review compared with the matched cohort. Highlighting this disparity is important because of the potential missed opportunities for skin cancer screening and tailored photoprotection advice. Although national skin cancer screening advice varies, ranging from biannual initially, annually or determined by individual needs and risk factors, a recent UK national study found that only 55% of transplant centres provided post-transplant skin cancer screening.^13–15^ Like many health inequalities, disparities in dermatological care among KTRLHIV, compared with HIV-negative KTR, may result from various factors including patient education, literacy and perception, healthcare professionals’ attitudes and implicit biases, and access to healthcare.^16,17^ This relatively lower rate of formal dermatology clinic assessment and review may have contributed to the significantly lower rates of photoprotection and skin cancer awareness in our cohort of KTRLHIV. Addressing these disparities by incorporating strategies to overcome barriers is crucial, as early detection and treatment of skin cancer rely on regular informed patient self-examination and dermatology surveillance. For example, through enhancing patient and healthcare provider skin cancer education and addressing implicit biases, access to dermatological care within pre-existing transplant clinics, community outreach, standardization of sustainable policies, funding and systemic change for continual dermatological care for patients who receive transplants.^16,18^

The majority of participants in our KTRLHIV cohort were Black (70%), which may be due to the demographics of South East London. A greater proportion of Black KTRLHIV were not reviewed by a dermatologist, denied receiving skin cancer awareness and photoprotection advice, did not partake in photoprotection behaviours and were not aware of their increased skin cancer risk. Ethnic disparities in skin cancer risk exist, with Black individuals demonstrating a reduced susceptibility to UV-related nonmelanoma skin cancers. However, rates of skin cancer in transplant recipients of colour were reported to be 6.9–12.5% in a multicentre retrospective study.^19^ Also, in a South African Black cohort of participants living with HIV, an odds ratio of 2.6 was noted for SCC.^20^ Moreover, due to lack of awareness and recognition, skin cancer diagnosis is often made at a more advanced stage and associated with greater morbidity and mortality.^21^ This introduces an emerging layer in the photoprotection and skin checking discourse, and should prompt reconsideration of screening measures based on the unique risk profiles associated with different ethnic backgrounds.^22^ Understanding this nuance is essential for developing tailored photoprotection and skin cancer awareness recommendations that align with the distinct characteristics of diverse patient populations.

Awareness of the increased risk of risk skin cancer was only 48% among KTRLHIV and 64% among HIV-negative KTR, a range akin to that reported in other single-centre studies.^23,24^ However, while select studies have reported high skin cancer awareness rates in transplant recipients, reaching as high as 91%, this awareness has not consistently translated into proactive photoprotection or routine skin-checking behaviours among their respective cohorts.^12,25,26^ A qualitative study employing the Health Belief Model explored this dissonance in skin cancer knowledge and lack of corresponding action. It reported that transplant recipients assigned their skin health a low priority rank as they did not perceive skin cancer to be significant health problem.^27^ Similarly, our qualitative analysis revealed many misconceptions in risk factors and consequences of skin cancer, and a sense of invulnerability through believing they were unlikely to develop skin cancer. The underlying behavioural psychology underpinning this requires further exploration, especially in cohorts such as KTRLHIV, who arguably contend with ranking a greater multitude of health problems, including navigating HIV control, medication interactions and concerns over increased rates of transplant rejection.^28,29^ There is a clear necessity for effective sun protection education highlighting the gravity of skin cancer, with the goal of translating this to altered behaviour, especially for high-risk groups, including KTRLHIV, and influencing post-transplantation skin cancer incidence.

There are currently no specific guidelines or consensus recommendations for the best times to provide photo­protection, skin cancer and self-skin examination education for KTRLHIV. Several studies have investigated the effectiveness of offering photoprotection and skin-checking guidance before transplantation, shortly thereafter or after an extended period, while also examining the frequency of advice delivery.^30–38^ While results vary across studies, there is consistent evidence indicating that education provided as a one-time event or during stressful periods (e.g. immediately before/after transplantation) is often insufficient. Repeated reinforcement of multimodal culturally sensitive education, involving follow-up appointments, written and multimedia resources, at 6-month intervals, has emerged as the most effective method.^39,40^ In addition, certain studies have indicated that offering sun protection education at the onset of summer, coupled with tailored reminders adjusted for prevailing weather conditions facilitated the adoption of sun protection practices.^39^ However, this can be resource-­intensive and was only conducted over a short period of time, failing to ascertain long-term behavioural change. Alternatively, some studies have advised risk-stratifying organ transplant recipients to then consider more intensified prevention strategies and dermatological care for this cohort.^41^

This study offers valuable insights into an under-­investigated population. However, limitations include the relatively small sample size and single-centre design, limiting the generalizability of the conclusions made from the study. Despite this, the KTRLHIV cohort size was significant relative to the cohort nationally. Also, the cross-­sectional design precluded longitudinal analysis of skin cancer outcomes, and the sample size was insufficient to assess significant differences in skin cancer incidence; further studies with larger cohorts are needed to explore this in detail. Additionally, self-reported data may be susceptible to recall bias and social desirability bias. Future studies of KTRLHIV with larger, multicentre prospective designs where various targeted educational interventions can be compared is ­warranted to confirm these findings and inform recommendations.

In conclusion, our study highlights significant disparities in dermatologist review, skin cancer awareness and photoprotection advice between KTRLHIV and HIV-negative KTR. Addressing these disparities through targeted interventions, including improved access to dermatology, equipping primary care and nephrology teams with skin cancer education, enhanced multimodal patient education, and tailored ­photoprotection and skin-checking strategies, is essential for optimizing skin cancer prevention and management in this high-risk population.

## Supplementary Material

vzaf016_Supplementary_Data

## Data Availability

The data underlying this article will be shared on reasonable request to the corresponding author.
