# Supplementary material for: Photoprotection and skin cancer awareness in kidney transplant recipients living with HIV: a single-centre cross-sectional study
Source: Skin Health Dis. 2025 May 12;5(4):256–62. doi: 10.1093/skinhd/vzaf016 (PMC12311170; doi:10.1093/skinhd/vzaf016)
Supplement: vzaf016_Supplementary_Data [file vzaf016_supplementary_data.docx]

# **Supplementary**

**Appendix S1** - *Photoprotection and Skin Cancer Awareness Questionnaire, adapted from Ismail et al.*

1. **Year of renal transplant**
2. **In the last year how often did you visit a kidney doctor?**
   Never / Once / Twice / Three times / Four or more times
3. **In the last year about how many times did you visit a skin doctor?**
   Never / Once / Twice / Three times / Four or more times
4. **Have you ever been given advice on how to protect your skin from sunlight?**
   Yes / No / Can’t remember
5. **From where did you get this advice on how to protect your skin from the sunlight?**

- Renal doctor: Yes / No / Can’t remember
- Skin doctor: Yes / No / Can’t remember
- Renal nurse: Yes / No / Can’t remember
- GP: Yes / No / Can’t remember
- Media: Yes / No / Can’t remember
- Other: Yes / No / Can’t remember (If yes, from where?)

1. **If you have received advice about protecting yourself from sunlight, when did you get this advice?**

- Before my first transplant: Yes / No / Can’t remember
- After my first transplant: Yes / No / Can’t remember

1. **How many times have you been given this advice?**
   Never / Only once / A few times / Often / Can’t remember
2. **Have you ever received written advice (e.g., a leaflet) about protecting yourself from sunlight?**
   Yes / No / Can’t remember
3. **Skin type:**
   I. Never tans, always burns
   II. Rarely tans, usually burns
   III. Usually tans, can burn
   IV. Always tans, rarely burns
   V. Asian/Middle Eastern
   VI. African/Afro-Caribbean
4. **Do you use sunscreen?**
   Yes / No
5. **If yes, how do you use it?**

- Only when sunny
- Daily all year
- Daily for part of the year

1. **If you use it daily, which months would you use it in the UK?**
   Jan Feb Mar Apr May June July Aug Sep Oct Nov Dec
2. **What factor sunscreen do you mainly use?**
   2–5 / 8–10 / 15–25 / more than 25
3. **Approximately how many tubes of sunscreen do you use each year?**
   None / 1 / 2 / 3 / 4 / more than 4
4. **Where do you apply sunscreen?**

- Face only
- Face and hands
- All exposed areas

1. **During the summer, do you specifically try and avoid being directly exposed to the sun?**
   Never / Sometimes / Usually / Always
2. **If you try and avoid being directly exposed to the sun, which times would you do this?**
   9 am/10 am/11 am/12 pm/1 pm/2 pm/3 pm/4 pm/5 pm/6 pm
3. **Do you dress to protect yourself against the sun (e.g., wear a hat)?**
   Never/Sometimes/Usually/Always
4. **Do you ever go on holiday to sunny countries?**
   Yes/No
5. **If yes, how often?**

- Once every 2–3 years
- Once a year
- More than once a year

1. **Why should transplant patients take extra precautions in the sun and regularly check their skin?** (Please answer in one or two sentences)
